# Supplementary material for: A fault-intrusion-tolerant system and deadline-aware algorithm for scheduling scientific workflow in the cloud
Source: PeerJ Comput Sci. 2021 Nov 2;7:e747. doi: 10.7717/peerj-cs.747 (PMC8576554; doi:10.7717/peerj-cs.747)
Supplement: Supplemental Information 6 [file peerj-cs-07-747-s006.zip › Myproject1.1/docs/org/cloudbus/cloudsim/package-summary.html]

org.cloudbus.cloudsim


JavaScript is disabled on your browser.


- Overview
- Package
- Class
- Use
- Tree
- Deprecated
- Index
- Help

- Prev Package
- Next Package

- Frames
- No Frames

- All Classes

# Package org.cloudbus.cloudsim

- Interface Summary

  | Interface | Description |
  | --- | --- |
  |  |  |
  | --- | --- |
  | Packet | This class contains the structure for a network packet. |
  | Storage | An interface which defines the desired functionality of a storage system in a Data Cloud. |
  | UtilizationModel | The UtilizationModel interface needs to be implemented in order to provide a fine-grained control over resource usage by a Cloudlet. |
- Class Summary

  | Class | Description |
  | --- | --- |
  |  |  |
  | --- | --- |
  | Cloudlet | Cloudlet is an extension to the cloudlet. |
  | CloudletScheduler | CloudletScheduler is an abstract class that represents the policy of scheduling performed by a virtual machine. |
  | CloudletSchedulerDynamicWorkload | CloudletSchedulerDynamicWorkload implements a policy of scheduling performed by a virtual machine assuming that there is just one cloudlet which is working as an online service. |
  | CloudletSchedulerSpaceShared | CloudletSchedulerSpaceShared implements a policy of scheduling performed by a virtual machine. |
  | CloudletSchedulerTimeShared | CloudletSchedulerTimeShared implements a policy of scheduling performed by a virtual machine. |
  | Consts | Defines common constants, used throughout cloudsim. |
  | Datacenter | Datacenter class is a CloudResource whose hostList are virtualized. |
  | DatacenterBroker | DatacentreBroker represents a broker acting on behalf of a user. |
  | DatacenterCharacteristics | DatacenterCharacteristics represents static properties of a resource such as resource architecture, Operating System (OS), management policy (time- or space-shared), cost and time zone at which the resource is located along resource configuration. |
  | DataCloudTags | This class contains additional tags for the DataCloud functionalities, such as file information retrieval, file transfers, and storage info. |
  | File | A class for representing a physical file in a DataCloud environment |
  | FileAttribute | A class for storing related information regarding to a `gridsim.datagrid.File` entity. |
  | HarddriveStorage | An implementation of a storage system. |
  | Host | Host executes actions related to management of virtual machines (e.g., creation and destruction). |
  | HostDynamicWorkload | The class of a host supporting dynamic workloads and performance degradation. |
  | HostStateHistoryEntry | The Class HostStateHistoryEntry. |
  | InfoPacket | InfoPacket class can be used to gather information from the network layer. |
  | Log | The Log class used for performing loggin of the simulation process. |
  | NetworkTopology | NetworkTopology is a class that implements network layer in CloudSim. |
  | Pe | CloudSim Pe (Processing Element) class represents CPU unit, defined in terms of Millions Instructions Per Second (MIPS) rating.  **ASSUMPTION: **All PEs under the same Machine have the same MIPS rating.**** |
  | ResCloudlet | CloudSim ResCloudlet represents a Cloudlet submitted to CloudResource for processing. |
  | SanStorage | SANStorage represents a storage area network composed of a set of harddisks connected in a LAN. |
  | UtilizationModelFull | The UtilizationModelFull class is a simple model, according to which a Cloudlet always utilize all the available CPU capacity. |
  | UtilizationModelNull | The UtilizationModelNull class is a simple model, according to which a Cloudlet always require zero capacity. |
  | UtilizationModelPlanetLabInMemory | The Class UtilizationModelPlanetLab. |
  | UtilizationModelStochastic | The UtilizationModelStochastic class implements a model, according to which a Cloudlet generates random CPU utilization every time frame. |
  | Vm | Vm represents a VM: it runs inside a Host, sharing hostList with other VMs. |
  | VmAllocationPolicy | VmAllocationPolicy is an abstract class that represents the provisioning policy of hosts to virtual machines in a Datacentre. |
  | VmAllocationPolicySimple | VmAllocationPolicySimple is an VmAllocationPolicy that chooses, as the host for a VM, the host with less PEs in use. |
  | VmScheduler | VmScheduler is an abstract class that represents the policy used by a VMM to share processing power among VMs running in a host. |
  | VmSchedulerSpaceShared | VmSchedulerSpaceShared is a VMM allocation policy that allocates one or more Pe to a VM, and doesn't allow sharing of PEs. |
  | VmSchedulerTimeShared | VmSchedulerTimeShared is a VMM allocation policy that allocates one or more Pe to a VM, and allows sharing of PEs by multiple VMs. |
  | VmSchedulerTimeSharedOverSubscription | This is a Time-Shared VM Scheduler, which allows over-subscription. |
  | VmStateHistoryEntry | The Class VmMipsAllocationHistoryEntry. |
- Exception Summary

  | Exception | Description |
  | --- | --- |
  |  |  |
  | --- | --- |
  | ParameterException | This exception is to report bad or invalid parameters given during constructor. |

- Overview
- Package
- Class
- Use
- Tree
- Deprecated
- Index
- Help

- Prev Package
- Next Package

- Frames
- No Frames

- All Classes
